# Two-in-One Sensor Based on PV4D4-Coated TiO_2_ Films for Food Spoilage Detection and as a Breath Marker for Several Diseases

**DOI:** 10.3390/bios13050538

**Published:** 2023-05-11

**Authors:** Mihai Brinza, Stefan Schröder, Nicolai Ababii, Monja Gronenberg, Thomas Strunskus, Thierry Pauporte, Rainer Adelung, Franz Faupel, Oleg Lupan

**Affiliations:** 1 Center for Nanotechnology and Nanosensors, Department of Microelectronics and Biomedical Engineering, Technical University of Moldova, 168 Stefan cel Mare Av., MD-2004 Chisinau, Moldova; mihai.brinza@mib.utm.md (M.B.); nicolai.ababii@mib.utm.md (N.A.); 2 Department of Materials Science, Chair for Multicomponent Materials, Faculty of Engineering, Kiel University, Kaiserstraße 2, D-24143 Kiel, Germany; ssch@tf.uni-kiel.de (S.S.); ff@tf.uni-kiel.de (F.F.); 3 Department of Materials Science, Chair for Functional Nanomaterials, Faculty of Engineering, Kiel University, Kaiserstraße 2, D-24143 Kiel, Germany; momo@tf.uni-kiel.de (M.G.); ra@tf.uni-kiel.de (R.A.); 4 Institut de Recherche de Chimie Paris—IRCP, Chimie ParisTech, PSL Université, 11 rue Pierre et Marie Curie, 75231 Paris, Cedex 05, France; thierry.pauporte@chimieparistech.psl.eu

**Keywords:** sensors, ammonia, hydrogen, PV4D4 polymer

## Abstract

Certain molecules act as biomarkers in exhaled breath or outgassing vapors of biological systems. Specifically, ammonia (NH_3_) can serve as a tracer for food spoilage as well as a breath marker for several diseases. H_2_ gas in the exhaled breath can be associated with gastric disorders. This initiates an increasing demand for small and reliable devices with high sensitivity capable of detecting such molecules. Metal-oxide gas sensors present an excellent tradeoff, e.g., compared to expensive and large gas chromatographs for this purpose. However, selective identification of NH_3_ at the parts-per-million (ppm) level as well as detection of multiple gases in gas mixtures with one sensor remain a challenge. In this work, a new two-in-one sensor for NH_3_ and H_2_ detection is presented, which provides stable, precise, and very selective properties for the tracking of these vapors at low concentrations. The fabricated 15 nm TiO_2_ gas sensors, which were annealed at 610 °C, formed two crystal phases, namely anatase and rutile, and afterwards were covered with a thin 25 nm PV4D4 polymer nanolayer via initiated chemical vapor deposition (iCVD) and showed precise NH_3_ response at room temperature and exclusive H_2_ detection at elevated operating temperatures. This enables new possibilities in application fields such as biomedical diagnosis, biosensors, and the development of non-invasive technology.

## 1. Introduction

Modern technologies are advancing every day, and with them the medical field and it’s diagnostic part, as well as the fields of health and food safety. To improve diagnosis, it is helpful to link the patients health states with data obtained from different health analyzing technologies. To improve diagnosis, it is helpful to link the patients’ health states with data obtained from different health analyzing technologies. In this regards, an interesting example of such an advance can be seen in a previous study [1] where a system of computer-aided diagnostics improved the results of a plain X-ray using machine learning. Another good example is an interpretable deep learning system in the study of Kai Jin et al. [2], where the main goal was to classify the epiretinal membrane for different optical coherence tomography devices, which, however, still needs further research, as its potential demonstrated. As a matter of fact, even previous and further studies, which will be seen in this work, have as their main goal minimal to non-invasive diagnosis, where much of the potential lies with gas detectors and sensors.

The introduction of novel gas detectors with rapid and efficient gas concentration detection capabilities has been a major focus for different application fields [3]. One of the current techniques that is being intensively developed is the breath test [4], which uses different methods, technologies, and analytical systems. These include different sampling injections methods and devices such as gas chromatographs (GC) but also sensor-based devices. While the GC might be a convenient method for breath analysis, it cannot detect H_2_ and is a rather expensive technique [5] compared to the field of fast-developing metal-oxide-based sensors. Metal-oxide sensors appear in many different forms, such as coated/uncoated with polymers [6], titanium carbide sensors [3], and many other compounds, e.g., titanium. Human breath contains many biomarkers, and it can show an entire series of different diseases and disorders [7,8,9,10].

However, there are not enough technologies and solid-state devices for the detection of these tracers, even though a recent approach to the gas detecting methods is surface plasmon resonance through optical means, where, for instance, a thin film of SnO_2_ and polypyrrole (PPy) were prepared for sensing ammonia [11]. In the same working field, another method for ammonia detection is shown in the study [12] through a colorimetric analysis is used to visualize manipulations of the localized resonance of the surface Plasmon band of silver nanoparticles. In this study, it was also shown that a smartphone can be used as a rapid, inexpensive method for real-time detection of ammonia by monitorin color intensity variations of an RGB analysis. In another study [13], a metal–organic framework was used as a colorimetric sensor for ammonia detection. On the other hand, metal-oxide-based sensors have yet to show their true potential and high efficiency through fast gas detection, as they are coated with polymers for adapting to different measurement conditions, therefore tuning up their properties. Many articles [5,7,8,9,10,14,15,16] offer a good base for further development of H_2_ gas and NH_3_ vapor in human breath detectors based on different sensors mostly because these two have shown a specific approach to diagnosis. For instance, H_2_ gas is usually associated with gastric disorders such as lactose intolerance and bacterial overgrowth within the small bowel and for diagnosing rapid passage of food through the small bowel [5], while in food industry H_2_ is usually mentioned as a spoilage factor to canned food [17]. NH_3_ vapor can usually be associated with kidney failure, which can be characterized at its early stages by detection of its concentration in exhaled breath. Another example of the use of NH_3_ detection is its recognition as a biomarker in the field of hepatic kidney diseases [18]. On the other hand, NH_3_ gas also serves as a spoiling marker for food rich in proteins [19]. Thus, further development of H_2_- and NH_3_-detecting sensors is required, as they provide a growing potential for enhanced detection and analysis in the biomedical diagnosis field.

While many authors are developing new methods for NH_3_, H_2_, and other vapor/gas detection [3,5,18], in this study, a sensor based on a TiO_2_ nanolayer fully covered with a Poly(1,3,5,7-tetramethyl-tetravinylcyclotetrasiloxane) (PV4D4) thin film is proposed as a two-in-one sensor with high potential for NH_3_ and H_2_ gas detection. The PV4D4 thin film on top of the sensor was fabricated by initiated chemical vapor deposition (iCVD) in the same way as in our previous study [6]. Attributed to its solvent-free nature and CVD-typical growth characteristics, the iCVD process enables a precise coverage of good-quality, tailored polymer nanolayers on the lower nanoscale on specimens with a large surface area or on more complex geometries [20,21] such as the TiO_2_ structures in this study. TiO_2_ has been proven in several articles as a compatible H_2_ detector. It shows a series of responses to different gases such as 2-propanol, n-butanol, ethanol, and acetone [22]. Consequently, the challenge is to maintain a high selectivity for H_2_. In another study, thin nano-sprayed layers of TiO_2_ show a variation in responses depending on the film thickness [23], having a high selectivity for H_2_ at 15 nm but without a clear response to NH_3_. At 20 nm thickness, it shows a better response to NH_3_ but is still lacking high selectivity towards H_2_. In this context, some authors have reported on the functionalization of the sensor with different noble metals such as Au [23], while others have coated sensors with a conductive polymer layer [24]. Our previous study [6] showed impressive results of the influence of iCVD-deposited PV4D4 thin films and their influence on the sensor performance. It can improve the selectivity for different gases regarding different structures. 

The motivation to use a PV4D4-coated TiO_2_ gas sensor in this study is to demonstrate a potential two-in-one sensor and its protection from ambient and efficiency. The developed two-in-one sensors exhibit high selectivity for certain gases at relatively low operating temperatures and high selectivity for other gases at higher operating temperatures. Since the applied polymer layer on top of the TiO_2_ films shows an effect on the selectivity of H_2_ and NH_3_, depending on the working temperature, it can be applied as a potential two-in-one sensor for breath analysis. Although further studies on different biomarkers related to different diseases and disorders are needed, the proposed sensor can provide new pathways in the field of medical diagnosis and the development of non-invasive technology.

## 2. Materials and Methods

### 2.1. Sample Production

TiO_2_ nanolayers were spray-pyrolysis grown on the surface of a glass substrate, as described in reference [23]. Next, all specimens were placed on a thermal heating plate, where we maintained the temperature at 450 °C for 25 min before the start of the spray-pyrolysis experiment, as mentioned previously by Pauporté et al. [23,25]. For the spray-pyrolysis solution, we used 7.1 mL isopropanol, 0.62 mL of titanium(IV) isopropoxide (TTIP), and 0.41 mL of acetylacetone in a mixture. The carrier ambient involved was an air flux, which was selected, as earlier reported, to gust the mixed aerosol directly onto the top of specimen, which was kept on the heated hot surface at 450 °C during the entire spray process [23,25]. The spray process of TiO_2_ layers was followed by a post-growth thermal annealing at 610 °C for 60 min in air.

After the TiO_2_ gas sensor fabrication and thermal treatment, a 25 nm PV4D4 nanolayer was grown on top of the sensors. The sensors were transferred to a iCVD reactor reported elsewhere [6,26] and custom built for this purpose. The reactor was evacuated by a rotary vane pump (Duo 10, Pfeiffer Vacuum). For the PV4D4 thin film deposition, flowrates of 0.2 sccm 1,3,5,7-tetramethyl-tetravinyl cyclotetrasiloxane (V4D4, Abcr, 97%) and 0.1 sccm Perfluorobutanesulfonyl fluoride (PFBSF, Chempur, 95%) were introduced to the reactor via needle valves. A process pressure of 40 Pa was maintained by a butterfly valve (615, VAT). The pressure was monitored by a capacitive manometer (Baratron, MKS instruments). In order to facilitate monomer adsorption on the samples, the specimen stage was heated to 32 °C by a thermostat (K6, Huber). A power of 44 Watts was applied to a NiCr filament array inside the reactor to start the deposition process. Additional PV4D4 thin films were also deposited on a silicon wafer for further chemical characterization as reference samples.

### 2.2. Computational

Similar to our previous study, the D4 molecule was chosen as a representative to estimate the dimension of the cyclotetrsiloxane rings in the PV4D4 polymer. The octamethylcyclotetrasiloxane (D4) molecule was first edited and pre-geometry optimized using a molecular editor (Avogadro 1.2). The applied force field was a universal force field (UFF). Density functional theory (DFT) was performed with the molecules to predict the molecular distances. For this purpose, another geometry optimization was performed via NWCHEM [27]. The geometry optimization was performed on the B3LYP/cc-pVDZ level and on the B3LYP/6-31G* level. The final results were illustrated and analyzed by a molecular visualizer (Jmol 14.31.34).

### 2.3. Sample Characterization

Fourier-transform infrared (FTIR) spectra were recorded using a FTIR spectrometer (Invenio-R, Bruker, Billerica, MA, USA) in transmission mode. The area from 400 cm^−1^ to 4000 cm^−1^ was measured at 4 cm^−1^ step width and 32 scans. The recorded spectra were b-spline baseline corrected using a graphic software (Origin Pro 2017, OriginLab, Northampton, MA, USA). The top surface of the PV4D4/TiO_2_ films was measured using scanning electron microscopy (SEM) within a REM-ZEISS device at a voltage of 3 kV to avoid the charging effect and a current of 1 μA. A spectrophotometer was used to characterize the optical properties of the heterojunctions. For X–ray diffraction (XRD) measurements, (a Seifert 3000 TT device, Ahrensburg, Germany) operated at a voltage of 40 kV and a current of 40 mA was used, as reported previously [28]. The gas response of the samples was measured by using the setup and protocol described previously using a computer-controlled Keithley2400 source-meter, Cleveland, OH, USA [23,29]. 

## 3. Results and Discussion

### 3.1. Characterization of the Fabricated Sensors

**Figure 1:**
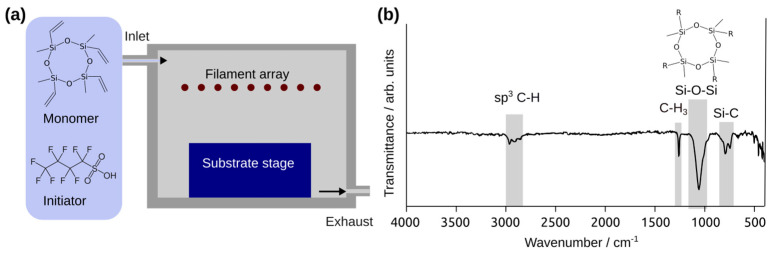
(**a**) Illustrative graphic of the iCVD process used in this study to deposit PV4D4 thin films on top of the TiO_2_ films-based sensors. (**b**) FTIR spectroscopy results for the deposited PV4D4 nanolayers.

The deposition process of the PV4D4 thin films via iCVD on top of the TiO_2_-based sensor structures is schematically illustrated in Figure 1a. 

**Figure 2:**
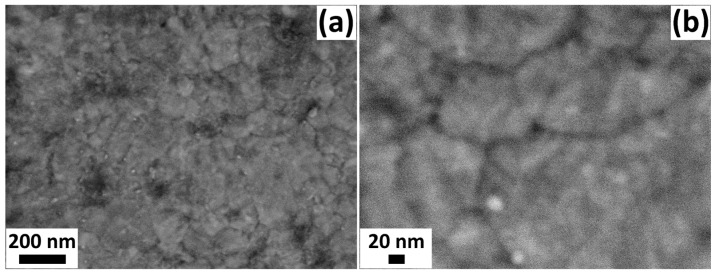
SEM images of the TiO_2_ structure coated with PV4D4 polymer nanolayer: (**a**) low magnification and (**b**) higher magnification.

The underlying reaction in iCVD is a free radical polymerization [30,31]. V4D4 monomer vapor and PFBSF initiator vapor are introduced into the reactor. Free radicals are generated by pyrolysis of the initiator molecules at the heated filament array. The V4D4 monomer molecules adsorb at the cooled substrate stage, i.e., the surface of the gas sensors. The free radicals meet the vinyl groups of the monomer and initiate a chain growth polymerization by connecting additional V4D4 molecules via the vinyl groups. Since V4D4 exhibits four vinyl groups, a highly crosslinked film is obtained after the polymerization. After the deposition, Fourier-transform infrared (FTIR) spectroscopy is performed in order to confirm a successful polymerization and preservation of the cyclotetrasiloxane rings of the V4D4 monomer molecules. The result is shown in Figure 1b. Successful polymerization of the V4D4 molecule is indicated by the absence of bands above 3000 cm^−1^ for the C-H stretch at a vinyl group as well as presence of bands at 2964 cm^−1^, 2905 cm^−1^, and 2864 cm^−1^, which indicate C-H3 methyl, C-H2, and C-H2, respectively [32]. The preservation of the cyclotetrasiloxane ring structure of the V4D4 monomer is shown by the strong band at 1058 cm^−1^, which corresponds to the Si-O-Si siloxane stretch in the form of cyclosiloxanes [33]. The observed bands indicate a successful polymerization as well as preservation of the monomer’s functionality. The thermal stability of iCVD cyclosiloxane-based polymer thin films has already been reported in one of our previous works. The fabricated polymer films are thus well suited for the application under the higher operating temperatures required for the metal-oxide gas sensors. It can protect the surface from a humid atmosphere as well as contribute to control the gas response to various species. The CVD growth enables a conformal coverage of large-area substrates and complex geometries, such as the TiO_2_ gas-sensing structures used in our study. The TiO_2_ structures coated with PV4D4 polymer thin films demonstrate excellent adhesion to the glass substrate, even after a long period of time (more than one year). Figure 2 shows the SEM images of the TiO_2_ structure after the deposition of the PV4D4 thin films via iCVD at low magnification (Figure 2a) and high magnification (Figure 2b). 

**Figure 3:**
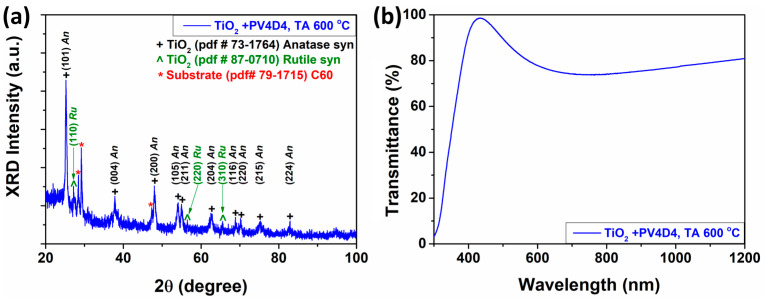
(**a**) The X-ray diffraction pattern and (**b**) UV–vis optical transmission spectra of the TiO_2_ with PV4D4 polymer.

**Figure A1:**
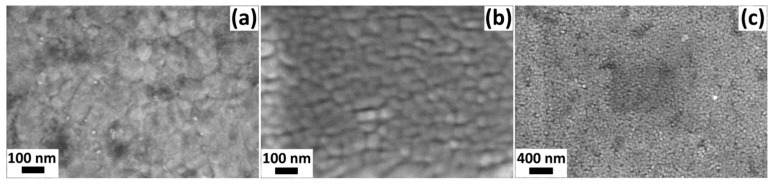
(**a**) SEM images of the TiO_2_ structure with PV4D4 polymer at scale of 100 nm; SEM images of the TiO_2_ structure without PV4D4 polymer at scale of (**b**) 100 nm and (**c**) 400 nm.

The TiO_2_ structures were thermally annealed at 610 °C for 1 h prior to the iCVD coating step. The images show that the structures are polycrystalline and made up of densely packed nanoparticles (see Figure A1b in the Appendix A). The TiO_2_ structures with the same thermal annealing regime but without polymer can be seen in Figure A1b. It is visible in this additional figure that the structures have a uniform, densely packed, granular morphology, which completely covers the glass substrate. The XRD pattern of the TiO_2_ structures fabricated with the PV4D4 polymer is shown in Figure 3a.

Two phases of titanium oxide were detected, namely the anatase phase and the rutile phase, which appear due to the thermal annealing regime at 610 °C, which was also demonstrated in other works [34,35,36]. The peaks detected at the 2θ angle positions 25.4°, 37.8°, 48.02°, 54.04°, 55.1°, 62.8°, 68.86°, 70.22°, 75.3°, and 82.9° correspond, according to the pdf card #73-1764, to the anatase phase with the corresponding Miller indices of (101), (004), (200), (105), (211) (204), (116), (220), (215), and (224), respectively. For the rutile phase, the 2θ peaks 27.24°, 56.46°, and 65.56° were detected, which, according to the pdf card #87-0710, correspond to the Miller indices (110), (220), and (310), respectively. In the same way, three other peaks were detected, which correspond to the substrate according to the pdf card #79-1715. Figure 3b represents the optical transmission spectrum of the TiO_2_ structures coated with PV4D4 polymer. The characteristic spectrum in the range of 300–1200 nm was performed. A high transparency in the order of 75% to 98% was observed over a wide range of wavelengths from 380 to 1200 nm. Besides the gas-sensing property, due to the high transparency of TiO_2_ structures, they are of great interest in real optical applications such as optical coatings [37], sunscreens [38], photocatalysis [39], and photodetectors [22,23]. The gas responses of developed sensors are shown in the next subsection. 

### 3.2. Gas-Sensing Measurements and Evaluation

**Figure 4:**
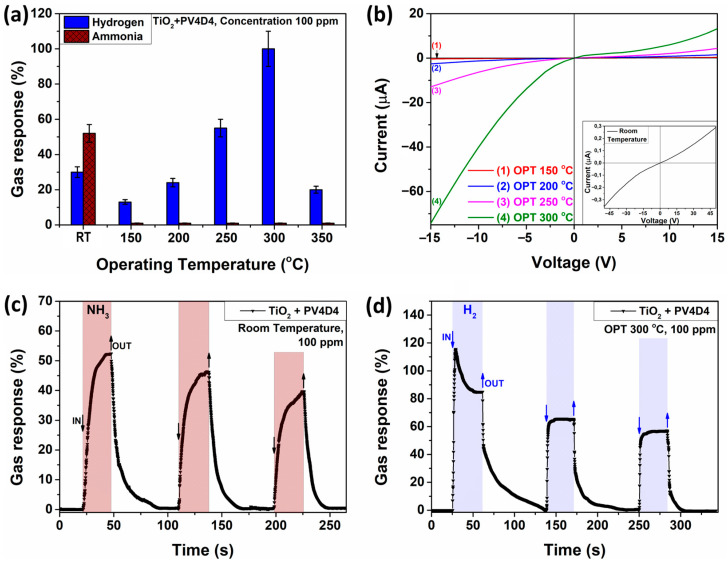
(**a**) Gas response of TiO_2_ structures thermally annealed at a temperature of 610 °C for 60 min and with PV4D4 polymer layer on surface-based sensor to 100 ppm of H_2_ gas and NH_3_ vapor at different operating temperatures; (**b**) current-voltage characteristics of the developed sensor, measured at different operating temperatures from 150 to 300 °C, and in insertion, it was measured at room temperature; (**c**) dynamic response to NH_3_ vapor of TiO_2_ films covered with PV4D4 polymer, measured at room temperature; (**d**) dynamic response to H_2_ gas of TiO_2_ films covered with PV4D4 polymer, investigated at operating temperature of 300 °C.

**Figure A2:**
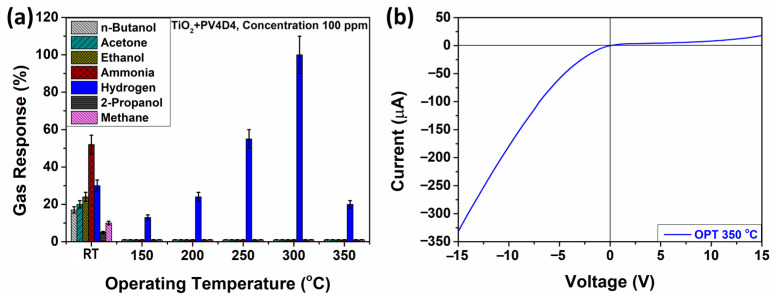
(**a**) Gas response versus operating temperatures for TiO_2_ sensor with PV4D4 coating on top to 100 ppm of gas/vapor (H_2_, NH_3_, n-butanol, acetone, ethanol, 2-propanol, and methane); (**b**) current-voltage characteristic at operating temperature of 350 °C of TiO_2_ covered with PV4D4 polymer and gold contacts.

The anticipated detection of H_2_ gas and NH_3_ vapor is investigated by gas-sensing measurements. The results are presented in Figure 4. Figure 4a shows the gas response of the PV4D4-coated TiO_2_ structures to H_2_ and NH_3_ at different operating temperatures. The measurement reveals that the operating temperature has an enormous impact on the selectivity of the sensor as well as the gas response. At room temperature, the sensor exhibits high NH_3_ vapor selectivity, with a response of ~52% for a concentration of 100 ppm. At higher temperatures, the selectivity changes towards H_2_ gas, also measured at a concentration of 100 ppm. The H_2_ gas response increases further for the operating temperatures of 250 °C and 300 °C. The highest response to H_2_ gas, showing a value of ~100%, is obtained at an operating temperature of 300 °C. The comparison of the results obtained for detection of NH_3_ and H_2_ with those in the literature are presented in Table 1 and Table 2, respectively. The gas responses of the PV4D4-coated TiO_2_ structures to NH_3_ vapor, H_2_ gas, and other types of vapors/gases that also are known and might be used as biomarkers in diagnosing certain diseases or have a certain degree of threat to health (n-butanol—marker between dementia with Lewy bodies and Parkinson’s disease [40]; acetone—diabetes [41]; ethanol—fatty liver disease [42]; 2-propanol—lung cancer determination [43]; methane—irritable bowel syndrome [44]) are represented in Figure A2a in the Appendix A. Figure A2a shows that besides NH_3_ vapor, a set of responses to acetone, n-butanol, methane, and ethanol is observed at room temperature. The selectivity is still dominated by NH_3_ vapor, which has the largest response at room temperature. In addition, Figure A2a reveals that at operating temperatures from 150 °C to 350 °C, the gas response is selective only to H_2_ gas. This observation can partially be attributed to the fabrication process of the TiO_2_. In our previous study, we showed for PV4D4-coated TiO_2_ sensors an almost 50% response at 300 °C. However, the TiO_2_ in our previous study was prepared by atomic layer deposition (ALD) without additional post-growth thermal annealing. This results in a different sensor performance compared to the TiO_2_ in this study, which is prepared by spray pyrolysis and subsequent thermally annealed at 610 °C for 1 h. This results in a different morphology and crystallinity, as already investigated in Figure 3a.

**Figure 5:**
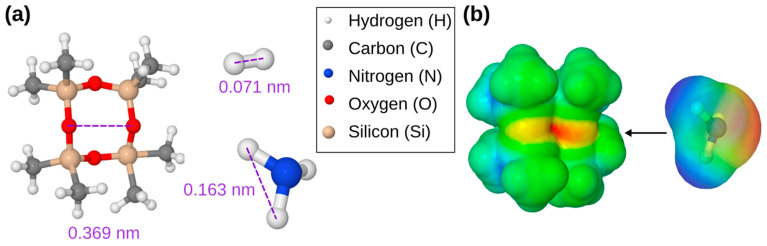
(**a**) Theoretically predicted distances within the D4 as well as H_2_ and NH_3_ molecules. (**b**) Calculated electrostatic potential maps for D4 and NH_3_ to illustrate their possible electrostatic interaction.

Figure 4b shows the current-voltage characteristics of the PV4D4-coated TiO_2_ sensors investigated at operating temperatures from 150 °C to 300 °C. With increasing temperature, a change in the current is observed, which was previously mentioned in a study [45] and attributed to the effect that is usually found in sensors based on metal oxides. The highest current from Figure 4b is determined at the reverse bias at 350 °C (curve 4). The current value reaches approximately 78 µA. The observed curves are very similar to the behavior of a Schottky diode or contact, observed in the early 1970s in the study of Lepselter, M.P. and Sze, S.M. [46]. The current-voltage characteristic at room temperature are included in the inset of Figure 4b. Although a range between −15 and +15 volts were chosen as the limits for the measurement, the inset for the measurement at room temperature is presented within the limits of −45 and +45 volts since the difference in an electrical current is very small, and a larger range allows more accurate observation of the manifestation of the characteristic curves. At room temperature, the current-voltage characteristic is almost linear. Additional volt-ampere characteristics of PV4D4-coated TiO_2_ structures at 350 °C operating temperature are represented in Figure A2b. The dynamic response of the PV4D4-coated TiO_2_ nanostructures to NH_3_ vapor with 100 ppm concentration measured at room temperature is shown in Figure 4c. For the first pulse, a response value of ~52% is observed. The response time is τ_r_ ≈ 12 s. The recovery time is determined to be τ_d_ ≈ 19 s. A repeated application of NH_3_ vapor indicates a small decrease in the response value and minimal influence on the response and recovery times. This can be explained by the fact that during the application of the second and third pulse, the sensor structure reaches saturation faster. Thus, the response is a little bit lower. Figure 4d evidences the dynamic response of the PV4D4-coated TiO_2_ structures to H_2_ gas at 100 ppm concentration and measured at a 300 °C operating temperature. During the first pulse, a sudden increase in the response value to H_2_ gas of ~115% is observed. Within a few seconds, a small decrease in the value follows, which is due to the physico-chemical effects that occur on the surface of the sensor structure. For the second and third pulse, a decrease in the response can be observed, similar to that shown in Figure 4c. This means there is a faster saturation of the sensor structure with H_2_ gas, while the effect of the first pulse already disappears. The response and recovery times are τ_r_ ≈ 3 s and τ_d_ ≈ 44 s, respectively. The transport of the respective gas molecules to the sensor surface can occur via the free volume and the cyclotetrasiloxane rings in PV4D4, as already suggested in our previous study [4]. Larger molecules such as organic solvents seem not to pass the cyclotetrasiloxane rings because they are simply too large. For this reason, hydrogen (H_2_) and ammonia (NH_3_) were investigated. In order to check if these molecules could potentially pass through the cyclotetrasiloxane rings, computational calculations are performed. The D4 molecule is used as a representative for the cyclotetrasiloxane ring structures present in the PV4D4 films. The presence of these rings is confirmed in the FTIR investigation of the PV4D4 already presented in Figure 1b. The D4 molecule is geometry optimized using density functional theory (DFT). In addition, the H_2_ and NH_3_ are geometry optimized in our calculations. The molecules and estimated dimensions of the molecules are shown in Figure 5a. 

The calculated distances in the molecules are 0.071 nm and 0.163 nm for H_2_ and NH_3_, respectively, which fits well with the literature values [47]. The cyclotetrasiloxane ring has a diameter of 0.369 nm according to our theoretical prediction. The H_2_ molecule is smaller, and it is more likely that it will travel via the ring. NH_3_ is also slightly smaller and could theoretically pass the ring. However, the molecular vibrations at elevated temperatures might significantly influence the pathways for the NH_3_. In addition, H_2_ is a non-polar molecule, while NH_3_ is a polar molecule, as shown by the electrostatic potential maps in Figure 5b. The polarity of NH_3_ might also lead to enhanced electrostatic interaction with the cyclotetrasiloxane ring, which provides four oxygen atoms per ring and, consequently, unpaired electrons. As shown in the measurement in Figure 4a, higher temperatures switch off the pathway for NH_3_ gas molecules. Thus, this might be traced back to increased molecular vibrations, which hinder the NH_3_ from penetrating through the polymer network as well as causing longer dwell times of the NH_3_ molecules at the cyclotetrasiloxane ring. In addition, electrostatic interaction might lead to a blocking of the NH_3_. The H_2_ molecules seems to be small enough to pass the polymer network. Compared to NH_3_ the H_2_ molecules are non-polar. This could additionally reduce the influence of electrostatic interaction accompanied by the free electrons of the oxygen atoms in the cyclotetrasiloxane ring. Thus, the selectivity of the sensor can be switched from ammonia for room temperature operating to hydrogen at higher temperatures above 150 °C. It can be applied as a potential two-in-one sensor for NH_3_ and H_2_, which can be controlled by the sensing temperature.

**Figure 6:**
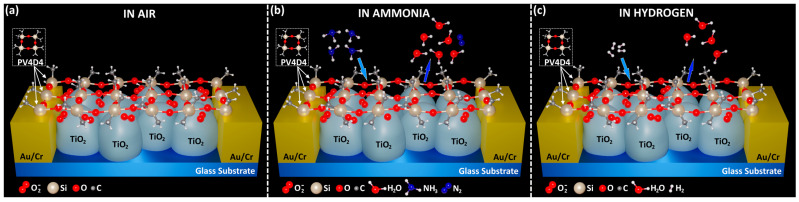
The proposed sensing mechanism of PV4D4-coated TiO_2_ nanostructures (**a**) in air; (**b**) in ammonia; (**c**) in hydrogen.

The sensing mechanism can be described by the effects that occur on the surface of the TiO_2_ structures. The selectivity to NH_3_ at room temperature and to H_2_ at relatively higher operating temperatures can be explained primarily due to the PV4D4 polymer layer by the fact that molecules such as organic solvents, etc., do not pass through the cyclotetrasiloxane rings due to their too-large dimensions, as described above. Secondly, this selectivity is due to the chemi-adsorption processes of oxygen species such as O_2_^−^, O^−^, or O^2−^ on the surface without the application of the test gas at different operating temperatures [22,29,48]. Thus, depending on the operating temperature, the oxygen in the ambient is adsorbed on the top surface of the TiO_2_ structure, which will lead to the extraction of free electrons and the formation of oxygen species (Figure 6a) as follows [49,50]:

(1) $$O_{2(gas)}↔O_{2(ads)}$$

(2) $$O_{2(ads)}+e^{-}\to O_{2(ads)}^{-}$$

(3) $$O_{2(ads)}^{-}+e^{-}↔{2O}_{\left(ads\right)}^{-}$$

(4) $$O_{\left(ads\right)}^{-}+e^{-}↔O_{\left(ads\right)}^{2-}$$

When applying the test gas, such as NH_3_ (Figure 6b), the redox reactions between the NH_3_ molecules and the oxygen species adsorbed on the surface take place according to the following relations [51]:

(5) $$NH_{3(gas)}↔{NH}_{3(ads)}$$

(6) $$4NH_{3}+{3O}_{2(ads)}^{-}\to {6H}_{2}O+{2N}_{2}+{3e}^{-}$$

(7) $$2NH_{3}+{3O}_{\left(ads\right)}^{-}\to {3H}_{2}O+N_{2}+{3e}^{-}$$

Following the reactions, H_2_O and N_2_ molecules are obtained with the release of electrons in the conduction channel of the TiO_2_ structure, thus increasing the response.

When H_2_ gas is applied, H_2_ molecules are adsorbed on the top surface of the TiO_2_ structure and will thus react with the adsorbed oxygen species, thus leading to surface reactions with the release of H_2_O (Figure 6c) and again to the release of electrons in the conduction channel of the TiO_2_ structure according to the following relationships [22,29,48]:

(8) $$H_{2}+\frac{1}{2}O_{2(ads)}^{-}\to H_{2}O+e^{-}$$

(9) $$H_{2}+O_{\left(ads\right)}^{-}\to H_{2}O+e^{-}$$

Thus, due to the PV4D4 polymer layer, we can reduce the selectivity of the structures only for molecules with dimensions up to 0.369 nm, e.g., NH_3_ and H_2_ in our case, and by directing the operating temperature, it is possible to change the selectivity between these two gases.

Table 1 represents the comparison of the results obtained for NH_3_, and Table 2 compares the results for H_2_ from this work with the results from the literature regarding the sensor materials used, which were coated/uncoated with different polymers.

**Table 1:** Comparison of the sensors (coated/uncoated with polymers) response to NH_3_.

Sensor Material	Polymer	Response, (%)	Concentration, (ppm)	Working Temp, (°C)
Graphene heterostructures [52]	Polypyrrole	45	10	RT
Ti_3_C_2_T_x_ films [53]	-	0.8	100	25
Co_3_O_4_ nanorod [54]	-	11.2	100	160
TiO_2_ films [22]	-	225 *	100	200
TiO_2_ films [23]	-	270 *	100	300
SnO_2_/polypyrrole nanocomposite [55]	Polypyrrole	57	0.1	RT
PPy/MnO_2_ composites [56]	Polypyrrole	3.79	100	RT
PPy-coated WO_3_ nanofibers [57]	Polypyrrole	6.3	1	100
NiO/PPy hybrid films [58]	Polypyrrole	246.6	350	25
**TiO_2_ (this work)**	**PV4D4**	**52**	**100**	**RT**

* Estimated from graphs.

**Table 2:** Comparison of the sensors (coated/uncoated with polymers) response to H_2_.

Sensor Material	Polymer	Response, (%)	Concentration, (ppm)	Working Temp, (°C)
TiO_2_ films [22]	-	600	100	250
TiO_2_ films [23]	-	640	100	300
SnO_2_ [59]	Teflon AF-2400	75	200	230
CuO/Cu_2_O films [48]	-	250 *	1000	350
TiO_2_/CuO/Cu_2_O films [48]	-	140	1000	350
**TiO_2_ (this work)**	PV4D4	100	100	300

* Estimated from graphs.

**Figure 7:**
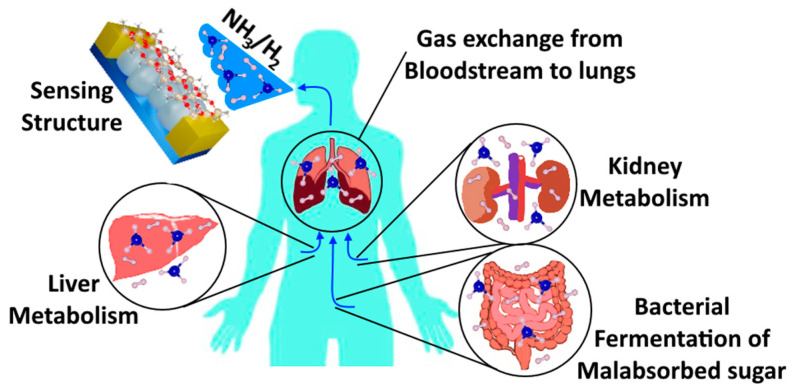
Concept of hydrogen and/or ammonia breath test.

The human body, through different mechanisms, daily releases different types of volatile organic compounds (VOCs) but also gases such as H_2_ and NH_3_, which may and eventually will be used in the medical diagnosis field. H_2_ gas is usually associated with gastric disorders, such as lactose intolerance and bacterial overgrowth in the colon, and with diagnosing rapid passage of food through small bowel [5]. Vast H_2_ gas in an organism is produced by bacterial fermentation of poorly absorbed sugar. NH_3_ gas, on the other hand, serves as a biomarker for kidney and liver metabolism, in which it can be detected if a patient might have hepatic kidney disease or kidney failure by different determined concentrations in exhaled breath [18]. While the whole process of the transfer of the mentioned biomarker may raise some questions, the mechanism of exchanging different VOCs/gases from the bloodstream to the exhaled breath through the lungs has already been studied for years. Therefore, information about it was already mentioned in 1982 by Johannes Piiper [60]. In the proposed research, the main concept is to use a solid sensing structure, such as a sensor, to catch the expired breath’s biomarkers and to determine by different concentrations the health conditions of patients. This step-in scientific progress will thus enable new possibilities in medical diagnosis. However, a vast experimental group for clinic trials is required to further determine the necessary concentration of different VOCs/gases and to elucidate relationships between them and further disorders. The investigated sensor is expected to be functional according to the concept presented in Figure 7. More details and findings will be presented in the forthcoming work.

**Figure 8:**
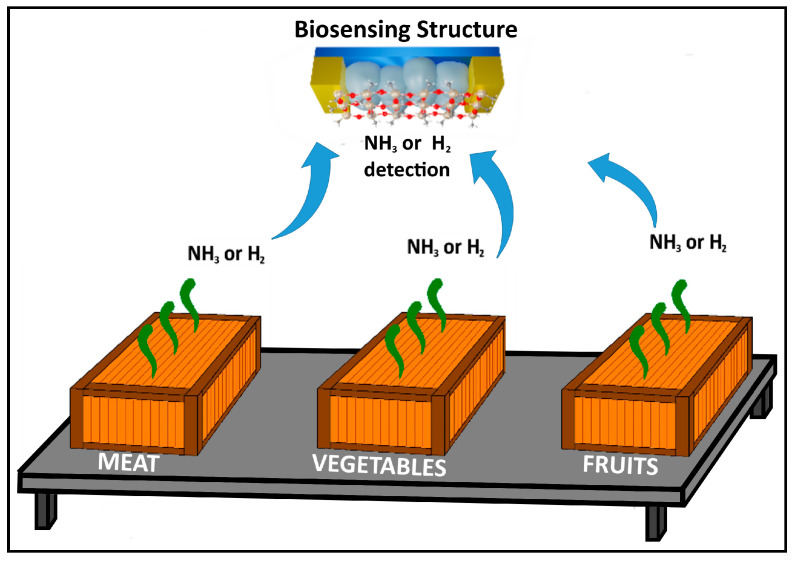
Concept of spoiled food detection.

While healthcare is mostly known for dealing with diseases, it must not be denied that the food industry has a large impact on health worldwide. A solution for tracking food safety may be finite credible data by block chain, which was studied by Li Yan et al. [61]; however, one of the most promising paths to resolving food waste and insuring the intake of quality food, which has an impact on health, may be a high-assurance method of determining if the food is spoiled or able to be consumed. Hanie Yousefi et al. [62] collected a series of parameters such as O_2_, CO_2_, temperature, humidity, pH change, and other specific chemicals that can be identified in the detection of a spoilage state of food in one or another period of time. NH_3_ usually is a marker gas for spoiling food, especially for products rich in proteins (e.g., eggs, meat, and dairy products), and therefore, this has been mentioned in similar works and confirmed in [19]. On the other hand, H_2_ is known to be a spoilage factor of canned food, which has been found to occur sometimes as the result of container deterioration. However, there are cases when deterioration results from the production of H_2_ from a reaction called rapid electrolytic detaining [17]. Still, research has yet to show what parameters and measurements of food quality may be attained through gas detection, as there are works such as [63] where the main target parameters are bioactive components of glutinous rice. For instance, in the work [64], through ultrasonic methods applied on dough, the CO_2_ volume showed changes. This parameter is yet to be researched and tested to understand the potential integration of gas detectors in the baking field. In the proposed research, a solid sensing structure otherwise known as a sensor is proposed to be used for sensing biomarkers such as NH_3_ and H_2_ to determine the quality of food. However, more experiments are required to determine the concentration of each marker for each type of food, and the main goal of the concept is to show the potential of such a sensor for use in the healthcare and food industries for better sustainability. Figure 8 show one of the concepts of spoiled food detection with such sensors.

## 4. Conclusions

A two-in-one sensor with a high potential for H_2_ and NH_3_ gas detection was developed based on new PV4D4-coated TiO_2_ nanostructures with two different crystal phases, i.e., rutile and anatase. XRD studies demonstrated the presence of these two phases after thermal annealing at 610 °C. The experimental results are supported by computational findings. They indicate a stable, precise, and very selective detector for the tracking of vapors at low concentrations. These developed sensors show a high selectivity for NH_3_ at room temperature. At higher temperatures above 150 °C, only H_2_ gas is detected by the presented sensor. This enables switching between NH_3_- and H_2_-sensing states by the operating temperature while using the same sensor. The detection of H_2_ is necessary to identify, e.g., the above-mentioned lactose intolerance, bacterial overgrowth of the small bowel, and rapid passage of food through small bowel. NH_3_ in breath is attributed to kidney failure and used as a biomarker in the field of hepatic kidney disease. Consequently, the demonstrated results can pave new paths in the field of biomedical diagnosis, i.e., breath detection, and development of non-invasive technology. The researched sensors might be used in clinical trials and could determinate reasonably accurate levels of NH_3_ and H_2_ from potential patients, therefore leading to more trials and results in developing the diagnosis field. As was stated, a certain threshold of different gases exhaled shows different tendencies towards different diseases. Nowadays, it is important to develop this direction to contribute to the development of healthcare, a process which will benefit everyone. On the other hand, healthcare, as discussed, is not only about diagnosing and healing certain diseases. One strong influence on human well-being is food and its quality. While quality might be understood as referring to the types of food that are humans supposed to intake, in this study, the quality of food refers to a state or not of spoilage, which is an important factor. A two-in-one sensor that can determine through detected gases the state of human health as well as the quality of food for ingestion and delivery to different distribution points offers a good base for further research as an instrument to more than one working field; as such, it has a high economic potential to be very accessible to the entire population. Although the economic factor was not the main point of the study, it cannot be neglected. In this regard, further studies are still necessary in the future, and therefore, this study is one of the many that will come to raise the awareness of diagnostic and food spoilage monitoring using hybrid-materials-based nano-scaled devices.

## Data Availability

Not applicable.
